# Wide Temperature 500 Wh kg^−1^ Lithium Metal Pouch Cells

**DOI:** 10.1002/anie.202503693

**Published:** 2025-05-20

**Authors:** Zichun Xiao, Xu Liu, Feng Hai, Yong Li, Duzhao Han, Xiangwen Gao, Zhenxin Huang, Yu Liu, Zhen Li, Wei Tang, Yuping Wu, Stefano Passerini

**Affiliations:** ^1^ School of Chemical Engineering and Technology Xi'an Jiaotong University Xi'an 710049 China; ^2^ School of Energy and Environment & Z Energy Storage Center Southeast University Nanjing 211189 China; ^3^ State Key Lab Space Power Sources Shanghai Institute Space Power Sources Shanghai 200245 China; ^4^ CNPC Tubular Goods Research Institute (TGRI) Xi'an 710049 China; ^5^ Future Battery Research Center Global Institute of Future Technology Shanghai Jiao Tong University Shanghai 200240 China; ^6^ Helmholtz Institute Ulm (HIU) Electrochemical Energy Storage Helmholtzstrasse 11 89081 Ulm Germany; ^7^ Karlsruhe Institute of Technology (KIT) P.O. Box 3640 76021 Karlsruhe Germany; ^8^ Center for Transport Technologies Austrian Institute of Technology (AIT) Giefinggasse 4 Wien 1020 Austria

**Keywords:** Electrolyte, Lithium metal batteries, SEI chemistry, Wide temperature adaption

## Abstract

The performance of lithium metal batteries is significantly affected by temperature variations, which makes it challenging for them to operate across a wide temperature range. Herein, a wide temperature adaption electrolyte is proposed, enabling excellent electrochemical performance of lithium metal batteries from −40 °C to 60 °C. Large, 5.8 Ah pouch cells employing such an electrolyte achieve high energy density of 503.3 Wh kg^−1^ at 25 °C with a lifespan of 260 cycles and outstanding energy density of 339 Wh kg^−1^ at −40 °C. The critical role of the solid electrolyte interphase (SEI) in determining the temperature‐dependent performance of lithium metal batteries is unveiled. It is demonstrated that the LiF‐rich, anion‐derived SEI facilitates Li^+^ diffusion in SEI. Moreover, accelerated Li^+^ desolvation at SEI is observed. These two aspects promote the kinetics of lithium metal anodes and further inhibit the dendrite growth at low temperatures. This work showcases the importance of understating the chemistry of SEI to enable wide temperature lithium metal batteries.

## Graphical abstract

The critical role of solid electrolyte interphase (SEI) in determining the temperature‐dependent performance of lithium metal batteries is unveiled. Through SEI chemistry regulation with a wide temperature adaption electrolyte, 5.8 Ah pouch cells achieve energy density of 503.3 Wh kg^−1^ at 25 °C with a lifespan of 260 cycles and outstanding energy density of 339 Wh kg^−1^ at −40 °C.

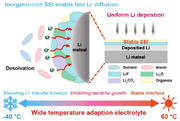

## Introduction

State‐of‐the‐art Li‐ion batteries have achieved great success in portable electronics and electric vehicles applications. Nevertheless, the limited theoretical energy density of Li‐ion batteries based on graphite anodes cannot satisfy the ever‐growing energy density demand. To this end, lithium metal batteries (LMBs) using high‐specific‐capacity lithium metal anode (3860 mAh g^−1^) have been regarded as the “Holy Grail” to achieve energy densities passing the 500 Wh kg^−1^ threshold.^[^ ^1^, ^2^, ^3^ ^]^ However, the lifespan of LMBs is compromised by the unstable metal electrode/electrolyte interface frequently causing dendritic lithium growth.^[^ ^4^ ^]^ Additionally, the performance of LMBs (as well as that of LIBs) is greatly affected by temperature variations. For instance, at low temperatures, the sluggish kinetics of lithium plating/stripping results in the Li dendrite growth and inferior discharge energy density (<300 Wh kg^−1^ at −40 °C).^[^ ^5^ ^]^ In contrast, at high temperatures, the unstable interface results in the continuous consumption of electrolyte and Li, leading to lifespan degradation.^[^ ^6^ ^]^ Therefore, LMBs also face limited operating temperature range challenges. To ensure the sufficient capacity and reliable lifespan of LMBs under low and high temperatures, external cooling and heating systems are commonly employed.^[^ ^6^ ^]^ However, introducing these systems inevitably increases the weight, the energy consumption and the manufacturing difficulty of the batteries, resulting in increased cost and decreased energy density at pack level. Therefore, it is necessary to extend the operating temperature range of LMBs.

The challenges brought by low temperatures mainly originate from slow Li^+^ transport kinetics,^[^ ^5^, ^7^, ^8^ ^]^ which have been categorized into two aspects. One is the hampered migration of Li^+^ due to increased electrolyte viscosity and even freezing at low temperatures. The other one is the high desolvation energy barrier of Li^+^. Significant efforts have been devoted to overcome these challenges, such as selecting low melting point solvents for a wide liquidus range to ensure sufficient ionic conductivity in bulk electrolyte^[^ ^9^, ^10^, ^11^, ^12^ ^]^ and constructing weakly solvating electrolytes (WSEs) by weakening interaction with Li^+^ to reduce the energy barrier of Li^+^ desolvation.^[^ ^13^, ^14^, ^15^ ^]^ These approaches mostly consider electrolyte design to elevate the Li^+^ transport kinetics. However, the solid electrolyte interphase (SEI) directly determines Li^+^ diffusion and Li dendrite growth.^[^ ^16^, ^17^, ^18^, ^19^ ^]^ Additionally, reducing the viscosity of the electrolyte by using low melting point solvents leads to stability issues at high temperatures, for example, 60 °C. Therefore, electrolyte engineering to enable LMBs operating across a wide temperature range still needs further exploration especially regarding its effect on SEI formation. SEI is, in fact, a critical aspect but received fewer attentions with respect to the bulk electrolyte region.^[^ ^3^, ^20^, ^21^ ^]^

Taking SEI chemistry and bulk electrolyte both into consideration, a wide temperature adaption electrolyte (WTAE) has been developed, which, by tuning the solvation structure from solvent‐dominated to anion‐dominated, enables the formation of inorganic‐rich SEI under low temperatures. The inorganic‐rich SEI decreases the activation energy for Li^+^ diffusion in the interphase layer and facilitates the Li^+^ desolvation at SEI, thus promoting the kinetics of lithium metal anodes and further inhibiting Li dendrite growth at low temperature. Consequently, WTAE enables LMBs to operate at a wide temperature range from −40 °C to 60 °C with superior electrochemical performance with respect to other electrolytes. Specifically, 5.8 Ah pouch cells achieve high energy density of 503.3 Wh kg^−1^ at 25 °C, with excellent stability over 260 cycles, and 339 Wh kg^−1^ at −40 °C.

## Results and Discussion

### Evaluation of Electrolyte in Wide Temperature Range

WTAE was prepared by dissolving 1 M lithium bis(fluorosulfonyl)imide (LiFSI) and 0.02 M LiNO_3_ in a 3:7 mixture (by volume) of 1,2‐dimethoxyethane (DME) and 1,1,2,2,‐tetrafluoroethyl‐2,2,3,3‐tetrafluoropropyl (TTE). DME was chosen as the solvating solvent due to its good compatibility with Li metal, low viscosity (0.45 cP at 25 °C), high solubility of LiFSI, and low freezing point (−58 °C). TTE served as the diluent solvent to overcome the high viscosity of highly‐concentrated electrolytes and inherit their anion‐dominated solvation structure.^[^ ^22^ ^]^ As shown in Figure S1, WTAE exhibited a wide liquidus range of −100 °C to 80 °C, making it feasible for wide temperature batteries.

**Figure 1:**
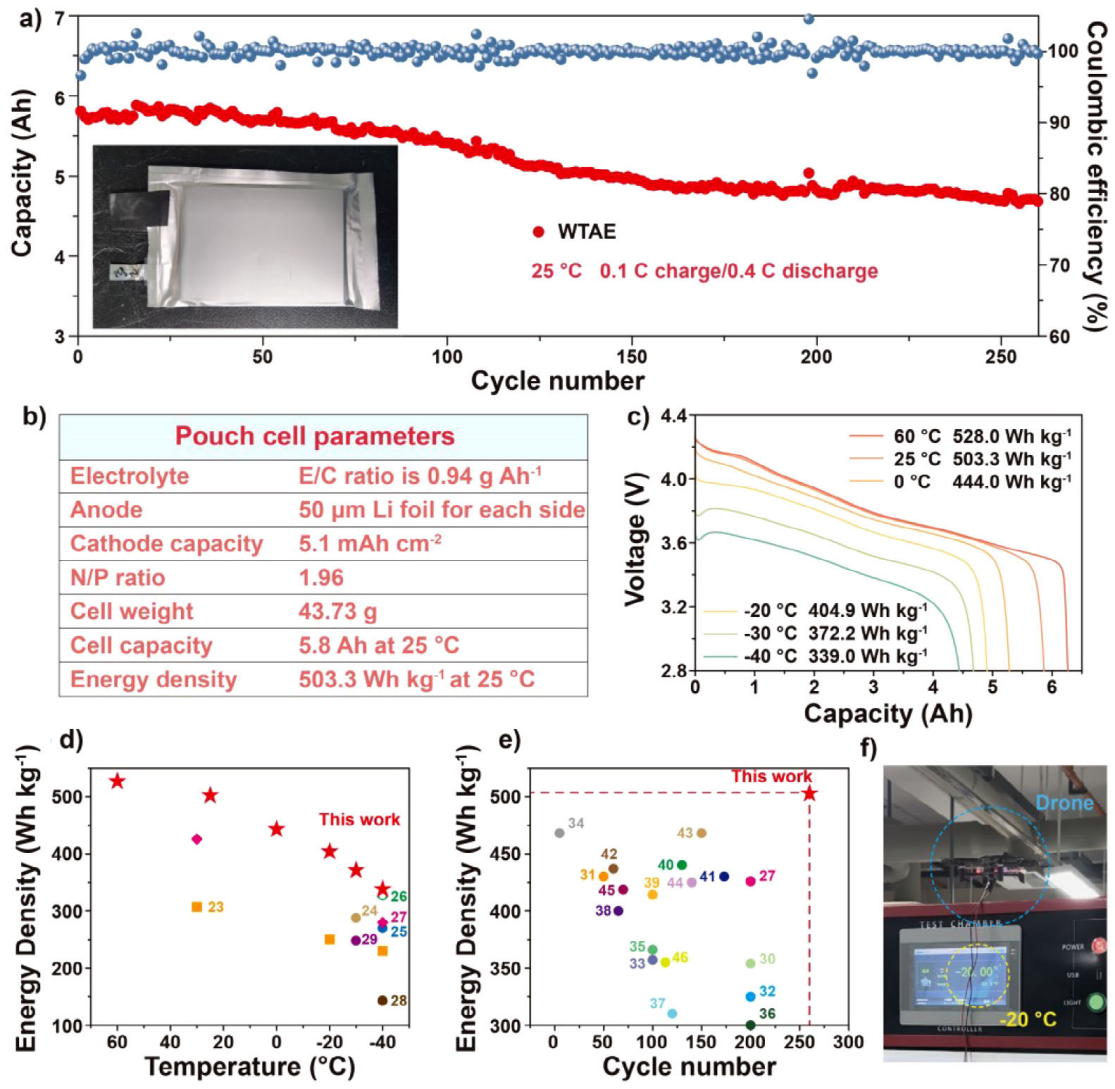
Evaluation of Li pouch cells with WTAE. a) Long‐term cycling of a 5.8 Ah pouch cell, the inset exhibits the optical image of the pouch cell. b) Specifications of the 5.8 Ah pouch cell. c) Voltage profiles of 5.8 Ah pouch cells discharged at various temperatures. d) Discharge energy density of LMBs at various temperatures reported in this work and in the literature. e) Energy density and cycle numbers of LMBs reported in this work and in the literature (criteria: energy density ≥ 300 Wh kg^−1^). f) Optical images of drones powered by the 1.28 Ah pouch cell discharged at −20 °C.

The wide temperature electrochemical performance of LMBs with WTAE was evaluated in both pouch cells and coin cells (Figures 1a and b and S3–S9, respectively). The 500 Wh kg^−1^ performance of a pouch cell employing WTAE under lean electrolyte conditions (E/C ratio is 0.94 g Ah^−1^) was first evaluated at room temperature. The energy density was calculated based on the total weight of the pouch cells, including packaging foil and tabs (details of the pouch cell specifications can be seen in Figure 1b and experimental section). Figure 1a shows the long‐term cycling performance of the 5.8 Ah pouch cell at a charge/discharge rate of 0.1 C/0.4 C and 25 °C, where the pouch cell achieved remarkable 80.6% capacity retention after 260 cycles. A few selected charge/discharge profiles upon cycling are displayed in Figure S2. The electrochemical performance of the 5.8 Ah pouch cell at sub‐ and super‐ambient temperatures was also evaluated. As shown in Figure 1c, the pouch cell can deliver across a wide temperature range from −40 °C to 60 °C with decent discharge energy density. Specifically, the discharge energy densities at −40, −30, −20, 0, 25, and 60 °C were 339.0, 372.2, 404.9, 444.0, 503.3, and 528.0 Wh kg^−1^, respectively. Figures 1d and e compare the energy density and lifespan performance of the 5.8 Ah pouch cell with other pouch cells reported in the literature, recently. The WTAE‐based pouch cell shows the highest discharge energy density at low temperatures.^[^ ^23^, ^24^, ^25^, ^26^, ^27^, ^28^, ^29^ ^]^ Additionally, it surpasses by far the gravimetric energy density and the cycling life of any other LMBs, achieving more than 500 Wh kg^−1^ and 250 cycles^[^ ^27^, ^30^, ^31^, ^32^, ^33^, ^34^, ^35^, ^36^, ^37^, ^38^, ^39^, ^40^, ^41^, ^42^, ^43^, ^44^, ^45^, ^46^ ^]^ (Figure 1e). As shown in Figure 1f, a WTAE‐based 1.28 Ah pouch cell operating at −20 °C successfully powered a drone for flight, demonstrating the practical application of LMBs with WTAE at low temperatures.

### Electrolyte Formulation Exploration

**Figure 2:**
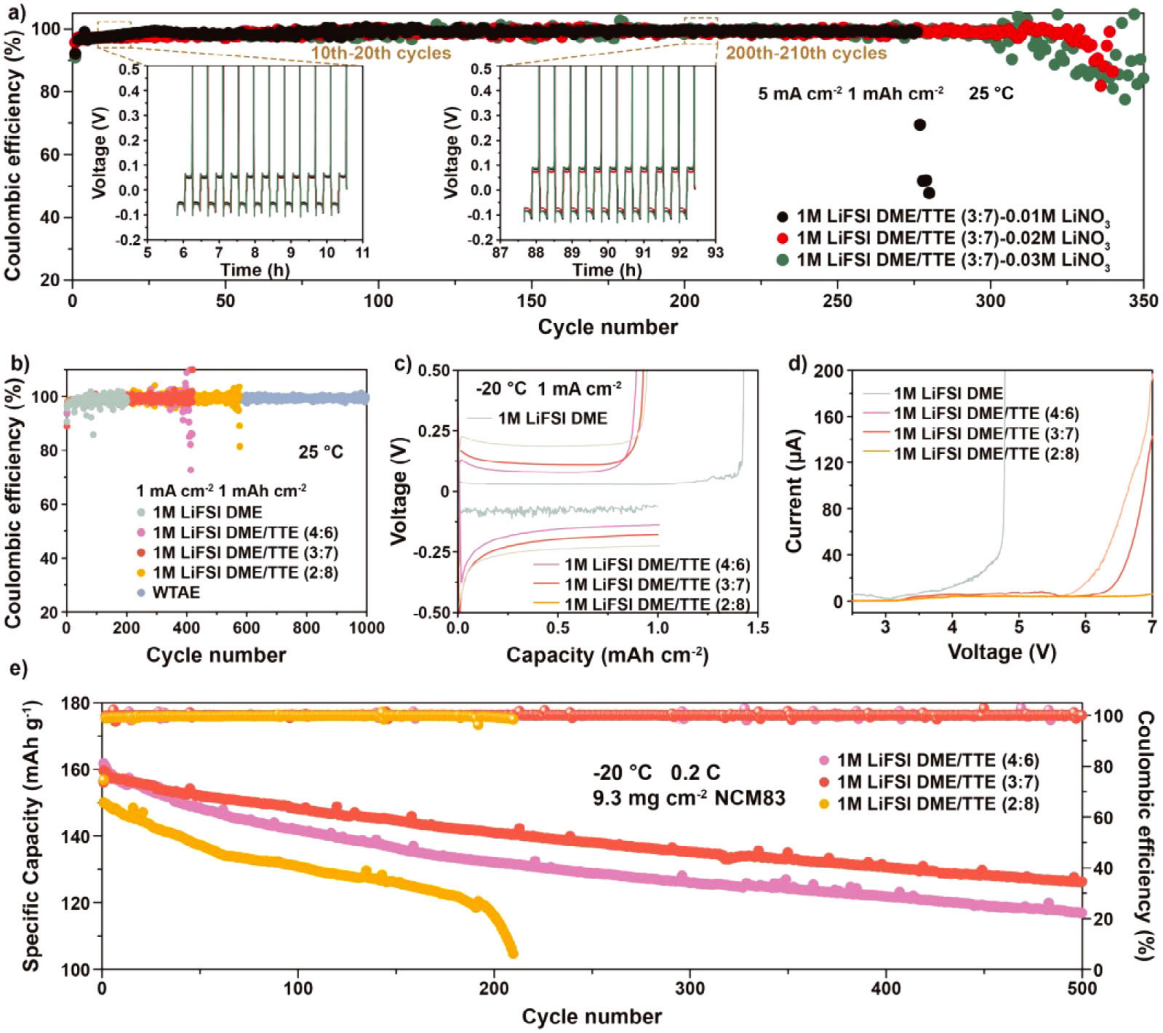
Explore the optimal electrolyte formulation. a) Coulombic efficiency of Li||Cu cells employing 1 M LiFSI DME/TTE (3:7) with different concentrations of LiNO_3_. b) Coulombic efficiency of Li||Cu cells employing WTAE and 1 M LiFSI DME/TTE electrolytes with different DME/TTE volume ratios. c) Discharge‐charge voltage curves of Li||Cu cells employing 1 M LiFSI DME/TTE electrolytes with different DME/TTE volume ratios. d) Anodic linear scan voltammetry profiles of 1 M LiFSI DME/TTE electrolytes over Super P coated Al current collector at 0.01 mV s^−1^. e) Long‐term cycling performance of Li||NCM83 cells employing 1 M LiFSI DME/TTE electrolytes with different DME/TTE volume ratios.

Electrolytes containing LiNO_3_ as additive are known to improve the electrochemical performance of LMBs.^[^ ^47^, ^48^, ^49^ ^]^ In the search for the optimal electrolyte formulation to meet the wide temperature requirements, the concentrations of LiNO_3_ as well as the solvent formulation were investigated. Figure 2a shows the performance of Li||Cu cells employing 1 M LiFSI DME/TTE (3:7) electrolytes with different LiNO_3_ concentrations upon long‐term cycling at 5 mA cm^−2^ current density and 1 mAh cm^−2^ areal capacity for each cycle. It can be observed that the electrolyte containing 0.02 M LiNO_3_ shows better cycling stability and lower polarization than those with 0.01 and 0.03 M. The cell overpotential is the smallest in the 0.02 M system, making this electrolyte more appropriate for application at low temperatures (Figure S10).

Subsequently, the effect of DME/TTE volume ratio on the performance of lithium metal anodes was investigated. Electrolytes with DME/TTE volume ratios from 1:0 to 2:8 were evaluated in Li||Cu cells employing a current density of 1 mA cm^−2^ and an areal capacity of 1 mAh cm^−2^ (for each cycle). As shown in Figure 2b, increasing the content of TTE in the electrolyte results in Li||Cu cells exhibiting better cycling stability. This derives from the formation of an inorganic‐rich SEI resulting from the shift towards an FSI^−^ dominated solvation of Li^+^ at highest TTE concentration in the electrolyte (Figures S11 and S12). The benefit is even more pronounced under lower temperatures as shown by the Li||Cu cells with different electrolytes tested at −20 °C. The voltage profiles at the 1^st^ cycle (Figure 2c) show that using 1 M LiFSI DME, large voltage fluctuations are observed while the plating capacity was even lower than the subsequent stripping capacity, supporting for the occurrence of short circuit and/or electrolyte decomposition. In contrast, the Li||Cu cells employing the DME‐TTE solvent mixtures show smooth profiles and high stripping/plating CEs, indicating that the ionic conductivity, the highest for 1 M LiFSI DME (Figure S13), is not the only factor determining the low‐temperature electrochemical performance of LMBs. The chemistry of SEI is certainly playing an important role.

DME owns poor electrochemical stability above 4 V versus Li/Li^+^,^[^ ^50^ ^]^ but the addition of TTE effectively promotes the oxidation stability of the mixed‐solvent electrolytes. This is confirmed by the linear sweep voltammetry (LSV) measurement (Figure 2d), where 1 M LiFSI DME/TTE electrolytes can withstand a high voltage of 5.5 V while 1 M LiFSI DME leads to a pronounced anodic current at 4 V.

**Figure 3:**
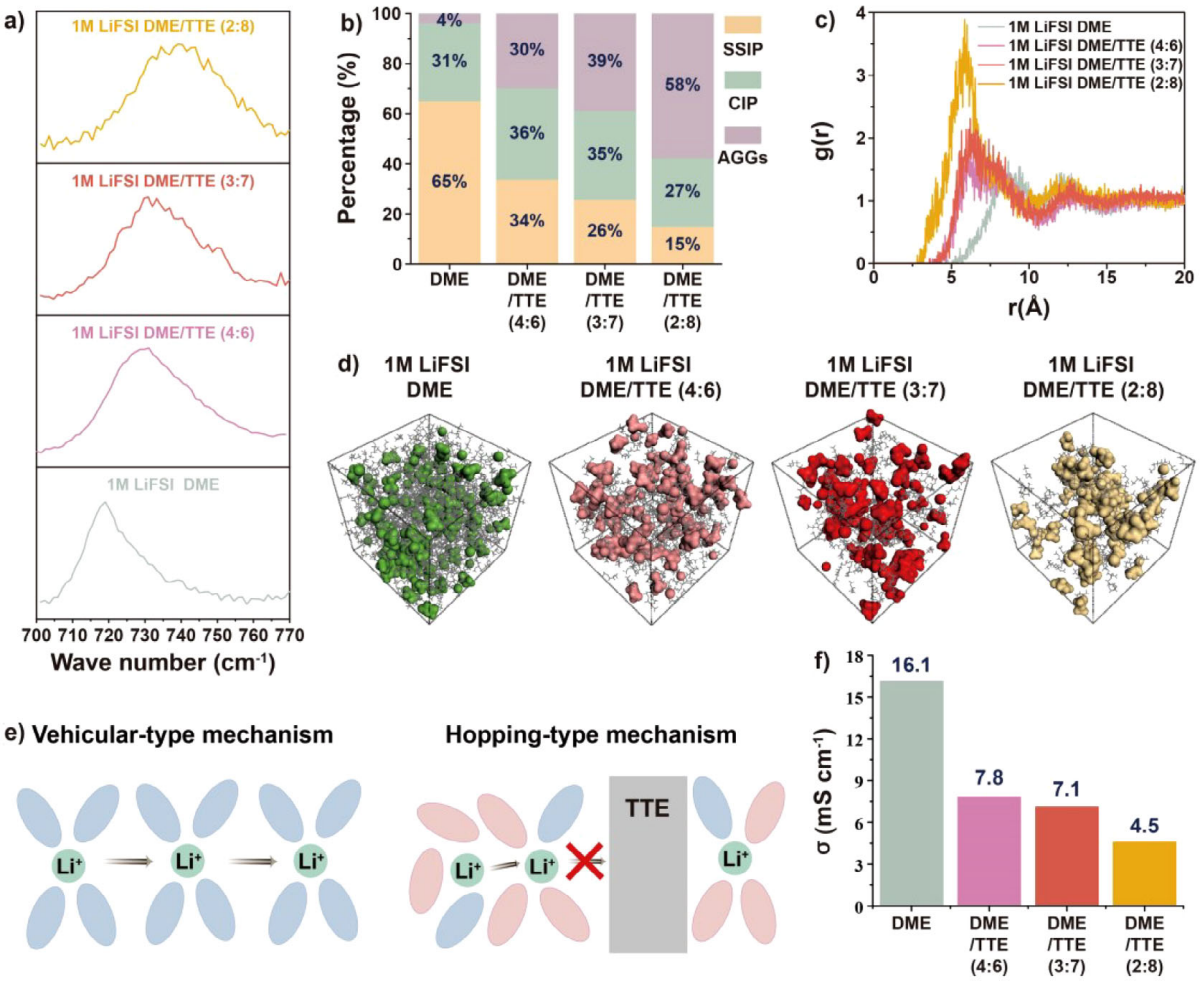
Solvation structure of 1 M LiFSI DME/TTE electrolytes with different DME/TTE volume ratios. a) Raman spectra of 1 M LiFSI DME/TTE electrolytes. b) Statistics of solvation structure distribution in 1 M LiFSI DME/TTE electrolytes. c) Radial distribution functions and coordination numbers of Li^+^‐Li^+^ in 1 M LiFSI DME/TTE electrolytes. d) Snapshots from the MD simulations of 1 M LiFSI DME/TTE electrolytes. Li^+^ and FSI^−^ are highlighted with different colors; DME molecules are presented as grey sticks and the non‐coordinating TTE molecules are set to invisible. e) Schematics of Li^+^ transportation mechanism. f) Measured ionic conductivity of 1 M LiFSI DME/TTE system electrolytes at 20 °C.

Further, the low‐temperature (−20 °C) electrochemical performance of LMBs with high areal loading NCM83 cathodes (9.3 mg cm^−2^) was evaluated. As shown in Figures 2e and S14, the Li||NCM83 cell with 1 M LiFSI DME/TTE (3:7) electrolyte exhibits a stable cycling behavior over 500 cycles with a capacity retention of 79.4%. Instead, the 1 M LiFSI DME/TTE (4:6) electrolyte leads to a lower capacity retention after 500 cycles while the 1 M LiFSI DME/TTE (2:8) electrolyte results in a quick cell failure after about 200 cycles. Thus, the optimal cycling performance of Li||NCM83 cells is achieved with the intermediate TTE content in the 1 M LiFSI DME/TTE electrolyte. To figure out the mechanism, the Li^+^ solvation structure in the 1 M LiFSI DME/TTE electrolytes was analyzed in more detail via Raman spectroscopy. As it can be observed in Figure 3a, the increased content of TTE leads to progressive shift of the S‐N‐S bending peak of FSI^−^ to high wavenumber, indicating that more FSI^−^ participate in the Li^+^ solvation shell. Besides, nuclear magnetic resonance (NMR) spectroscopy was also employed to validate the Li^+^ coordination environment. Coaxial NMR tubes were used with LiCl/D_2_O as an internal standard. As shown in Supplementary Figure S15, the increased concentration of TTE leads to a gradual upfield shift of the ^7^Li‐NMR peak. This is related to the shield effect of Li^+^, suggesting that more FSI^−^ enter into the Li^+^ solvation shell. In addition, this is also demonstrated by the statistics of SSIP (solvent‐separated ion pairs), CIP (contact ion pairs), and AGG (aggregates) proportion collected from the MD simulations (Figure 3b), indicating that more AGGs and less SSIP are formed with increasing TTE content. Also, as shown in Figure 3c, the distance between two Li^+^ ions becomes shorter. Therefore, it can be concluded that the increase of TTE content results in more AGGs, which tend to get closer to form compact large clusters as confirmed by MD simulations (Figure 3d), where Li^+^ and FSI^−^ are highlighted with different colors; DME molecules are presented as grey sticks and the non‐coordinating TTE molecules are set to invisible. It is noted that Li^+^ transport would transform from vehicular‐type to hopping‐type mechanism with increasing TTE contents as the molar ratio between lithium salt and solvating solvent, that is, DME, is increased.^[^ ^51^, ^52^ ^]^ However, very high fractions of the non‐solvating TTE could also obstruct the Li^+^ hopping conduction (Figure 3e),^[^ ^53^, ^54^, ^55^ ^]^ because the local domains of the Li^+^ solvated structures are separated by large TTE domains. This aspect, together with the reduced concentration of ionic charge carriers due to the formation of AGGs, leads to the ionic conductivity decrease of 1 M LiFSI DME/TTE with increasing TTE content (Figures 3f and S13). In fact, while the conductivity of the electrolytes with DME/TTE ratios of 4:6 and 3:7 show ionic conductivities above 7 mS cm^−1^, that of 1 M LiFSI in DME/TTE (2:8) electrolyte shows a sharp decline to only 4.5 mS cm^−1^. The inferior cycling performance of the Li||NCM83 cells with 1 M LiFSI DME/TTE (2:8) at −20 °C could be related to the significantly lowered Li^+^ transport in the electrolyte (see Figure S13).

### SEI Chemistry Analysis

**Figure 4:**
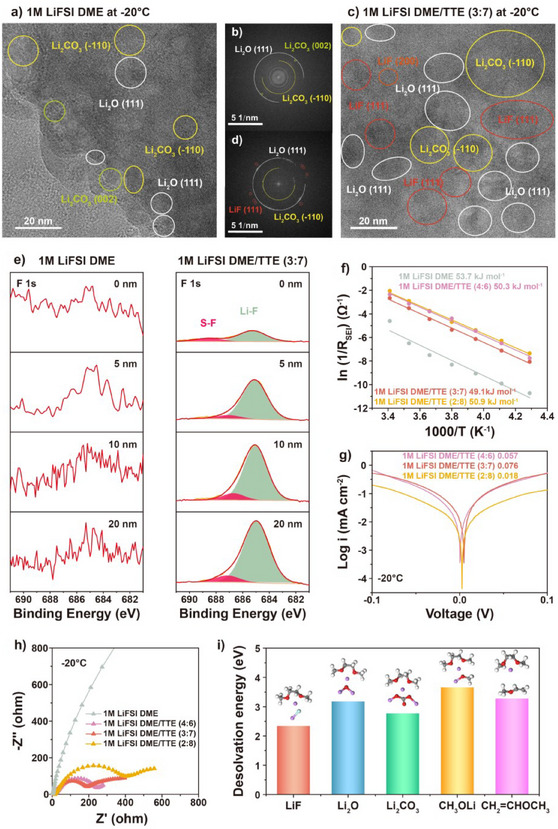
SEI composition and property. a) High‐resolution Cryo‐TEM image of the Li deposited in 1 M LiFSI DME electrolyte. b) Corresponding FFT pattern of a. c) High‐resolution Cryo‐TEM image of the Li deposited in 1 M LiFSI DME/TTE (3:7) electrolyte. d) Corresponding FFT pattern of c. e) F 1s XPS spectra of Li foils cycled in 1 M LiFSI DME and 1 M LiFSI DME/TTE (3:7) electrolytes with different Ar^+^ sputtering time. f) Activation energy of R_SEI_ fitted by the Arrhenius equation for 1 M LiFSI DME/TTE electrolytes. g) Tafel plots for Li plating/stripping in 1 M LiFSI DME/TTE electrolytes. h) EIS spectra of Li||Li symmetric cells 1 M LiFSI DME/TTE system electrolytes after 10 stripping/plating cycles. i) Desolvation energy barrier of the Li^+^‐DME solvation structure on different interface components. Balls with various colors represent different atoms: white, H; red, O; grey black, C; purple, Li; light blue, F.

As mentioned above, the SEI chemistry could be another key factor determining the electrochemical performance of LMBs at low temperatures. To understand the SEI chemistry effect on the low‐temperature performance, cryogenic‐transmission electron microscopy (Cryo‐TEM) was utilized to analyze the compositions of the SEI generated in 1 M LiFSI DME and 1 M LiFSI DME/TTE (3:7) electrolytes at −20 °C. The high‐resolution Cryo‐TEM image of the SEI formed in 1 M LiFSI DME (Figure 4a) shows inorganic crystallite of Li_2_O and Li_2_CO_3_ embedded in an amorphous organic layer. The presence of these inorganic species is further verified with the corresponding fast Fourier transform (FFT) pattern (Figure 4b). The high‐resolution TEM image of the SEI layer formed in 1 M LiFSI DME/TTE (3:7) electrolyte and the corresponding FFT pattern are displayed in Figure 4c and d, respectively. Besides Li_2_O and Li_2_CO_3_, LiF is also observed, which is attributed to the decomposition of the FSI^−^ anion. The absence of the LiF crystallite in the SEI generated in 1 M LiFSI in DME may explain the sharp difference in the low‐temperature performance between 1 M LiFSI DME and 1 M LiFSI DME/TTE (3:7) electrolytes.

In‐depth X‐ray photoelectronic spectroscopy (XPS) was further carried out as a complementary technique to investigate the SEI compositions formed at −20 °C. In the SEI derived from 1 M LiFSI DME electrolyte (Figures 4e and S16a), the signal of S‐O only existed at 0 nm and the signal of F was not detected. In sharp contrast, a strong signal of Li‐F and S‐O were detected in the SEI formed in 1 M LiFSI DME/TTE (3:7) electrolyte across a depth range of 0–20 nm,^[^ ^28^ ^]^ confirming the FSI^−^ decomposition to participate in the SEI formation.^[^ ^56^, ^57^ ^]^ In addition, in the C 1s spectrum (Figure S16b), the SEI derived from 1 M LiFSI DME electrolyte shows strong signal of R‐Li while the signal of C‐O_3_ only exists at 0 nm.^[^ ^28^, ^58^ ^]^ On the other hand, the signal of R‐Li is significantly lower in the 1 M LiFSI DME/TTE (3:7) electrolyte, indicating that the organic components are predominantly present in the outer SEI region. All of these indicate that 1 M LiFSI DME electrolyte tends to form a solvent‐derived SEI containing more organic components while the 1 M LiFSI DME/TTE (3:7) electrolyte tends to form an anion‐derived SEI, which is rich in inorganic components. The inorganic components in SEI could form abundant grain boundaries and vacancies, facilitating the Li^+^ diffusion due to lower diffusion energy barriers.^[^ ^59^, ^60^ ^]^The elements’ atomic ratios in the SEI reveal that the anion‐derived SEI from 1 M LiFSI DME/TTE (3:7) electrolyte consists of a LiF‐rich inner layer (Figure S17). The high surface energy of LiF‐rich inner layer can also effectively suppress the Li dendrite growth.^[^ ^61^, ^62^ ^]^ Hence, the superior low‐temperature electrochemical performance of 1 M LiFSI DME/TTE (3:7) electrolyte could be attributed to the anion‐derived SEI with a LiF‐rich inner layer.

The electrochemical performance of the cells with the solvent‐derived or the anion‐derived SEI were evaluated. The temperature‐dependent electrochemical impedance spectroscopy results (Figures S18–S21) were analyzed using the equivalent circuit shown in Figure S22. The activation energy for Li^+^ diffusion through the SEI (E_SEI_) was calculated according to the Arrhenius equation. As shown in Figure 4f, the E_SEI_ for 1 M LiFSI DME electrolyte is 53.7 kJ mol^−1^, while the E_SEI_ for 1 M LiFSI DME/TTE (4:6), (3:7), and (2:8) electrolytes is 50.3 , 49.1 , and 50.9 kJ mol^−1^, respectively. Moreover, the Tafel plots were acquired to evaluate the Li^+^ transfer kinetics at the electrode interface at −20 °C. As presented in Figure 3d, the exchange current density in 1 M LiFSI DME/TTE (4:6), (3:7), and (2:8) electrolytes is 0.057, 0.076, 0.018 mA cm^−2^, respectively, showing the fastest charge transfer kinetics at the SEI in the 1 M LiFSI DME/TTE (3:7) electrolyte. In addition, as shown in Figures S18–S21 though the resistance of the interfacial impedance (R_SEI_) in all electrolytes increases when the temperature decreases, the R_SEI_ in the 1 M LiFSI DME electrolyte is the highest, while the R_SEI_ in the 1 M LiFSI DME/TTE (3:7) electrolyte is the smallest at various temperatures, suggesting for higher Li^+^ diffusion occurring through the anion‐derived SEI. In addition, DFT calculations were applied to verify the role of different SEI components on the desolvation process. DME was chosen as the prominent coordinated molecule due to its strong solvation power compared to FSI^−^. As displayed in Figure 4i, the Li^+^ desolvation energy based on LiF is 2.34 eV, lower than that based on Li_2_O (3.18 eV), Li_2_CO_3_ (2.77 eV), CH_3_OLi (3.66 eV), and CH_2_CHOCH_3_ (3.28 eV). These results demonstrating that LiF‐rich SEI layer could facilitate the Li^+^ desolvation. In all, 1 M LiFSI DME/TTE (3:7) electrolyte exhibits lowest E_SEI_, lowest R_SEI_, and highest exchange density among all electrolytes, suggesting that SEI derived from 1 M LiFSI DME/TTE (3:7) electrolyte enables fast Li^+^ diffusion kinetics.

### Li Dendrite Growth Suppression

**Figure 5:**
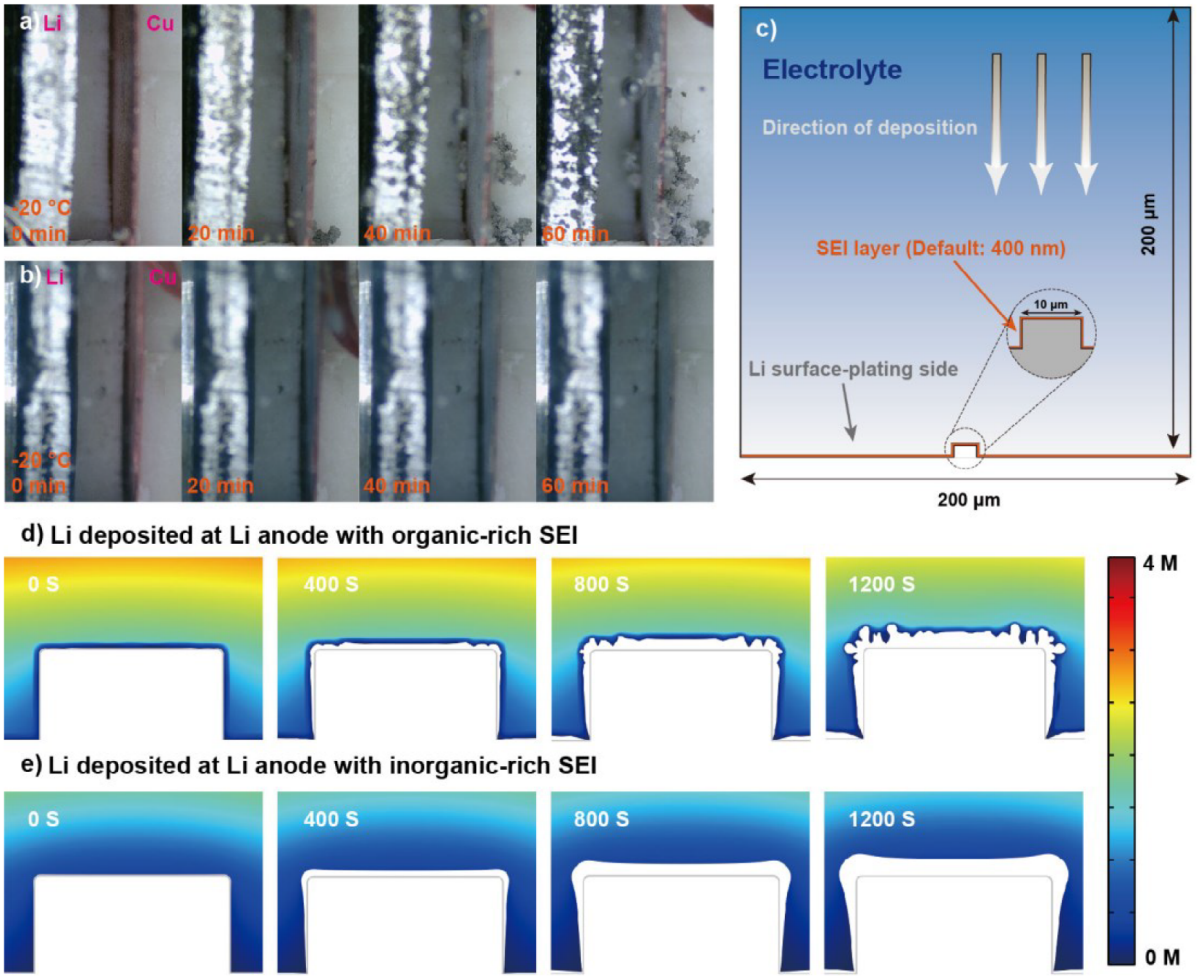
Suppression of Li dendrite growth. a) In situ optical observation of Li plating on the Cu foil with 1 M LiFSI DME electrolyte at −20 °C. b) In situ optical observation of Li plating on the Cu foil with 1 M LiFSI DME/TTE (3:7) electrolyte at −20 °C. c) Reference model used for simulation. d) Evolution of Li deposition behavior with organic‐rich SEI. e) Evolution of Li deposition behavior with inorganic‐rich SEI.

Following, the ability of SEI derived from 1 M LiFSI DME/TTE (3:7) electrolyte to inhibit Li dendritic growth was investigated. The Li deposition in 1 M LiFSI DME and 1 M LiFSI DME/TTE (3:7) electrolytes at −20 °C was observed by in situ optical microscopy. As shown in Figure 5a and Video S1, Li dendrites are observed within 20 min and rapidly grow and accumulate on the surface of the Cu foil in 1 M LiFSI DME electrolyte. By sharp contrast, in 1 M LiFSI DME/TTE (3:7) electrolyte, Li gradually covers the entire Cu foil, becoming thicker upon increasing deposition capacity (Figure 5b). Homogenous Li deposition is further confirmed even after 80 min (Figure S23). In addition, ex situ scanning electron microscopy (SEM) was applied to compare the Li deposition morphology at −20 °C in the two electrolytes. After deposition of 5 mAh cm^−2^, the morphology of Li deposits in 1 M LiFSI DME electrolyte exhibits prominent growth of whisker‐like Li dendrites, as is seen in the SEM image (Figure S24a). In contrast, the deposit from 1 M LiFSI DME/TTE (3:7) electrolyte shows a dense and granular morphology (Figure S24b). Furthermore, COMSOL Multiphysics simulation was conducted to investigate the Li initial growth with different SEI formation. The reference model is described in Figure 5c.

In this simulation, an initial protuberance was introduced on the center of Li metal electrode surface to induce Li nucleation and growth. Also, an organic‐ or inorganic‐rich SEI layer was set on the Li metal electrode. Using this model as the initial geometry under the collective driving force of electric field and ionic concentration gradient, Li begins to deposit at the protuberance. As illustrated in Figure 5d, the Li deposition with the organic‐rich SEI layer is uneven (400 s) and then gradually forms a number of branches after 1200 s. However, the morphology of the Li deposit with the inorganic‐rich SEI layer maintains uniformity and compactness (Figure 5e). As confirmed by in situ optical microscopy, ex situ SEM observations, and COMSOL Multiphysics simulation, the distinct Li deposition morphology strongly demonstrates that the anion‐derived SEI formed in 1 M LiFSI in DME/TTE (3:7) electrolyte can inhibit the Li dendrite growth, leading to a uniform Li deposition.

**Figure 6:**
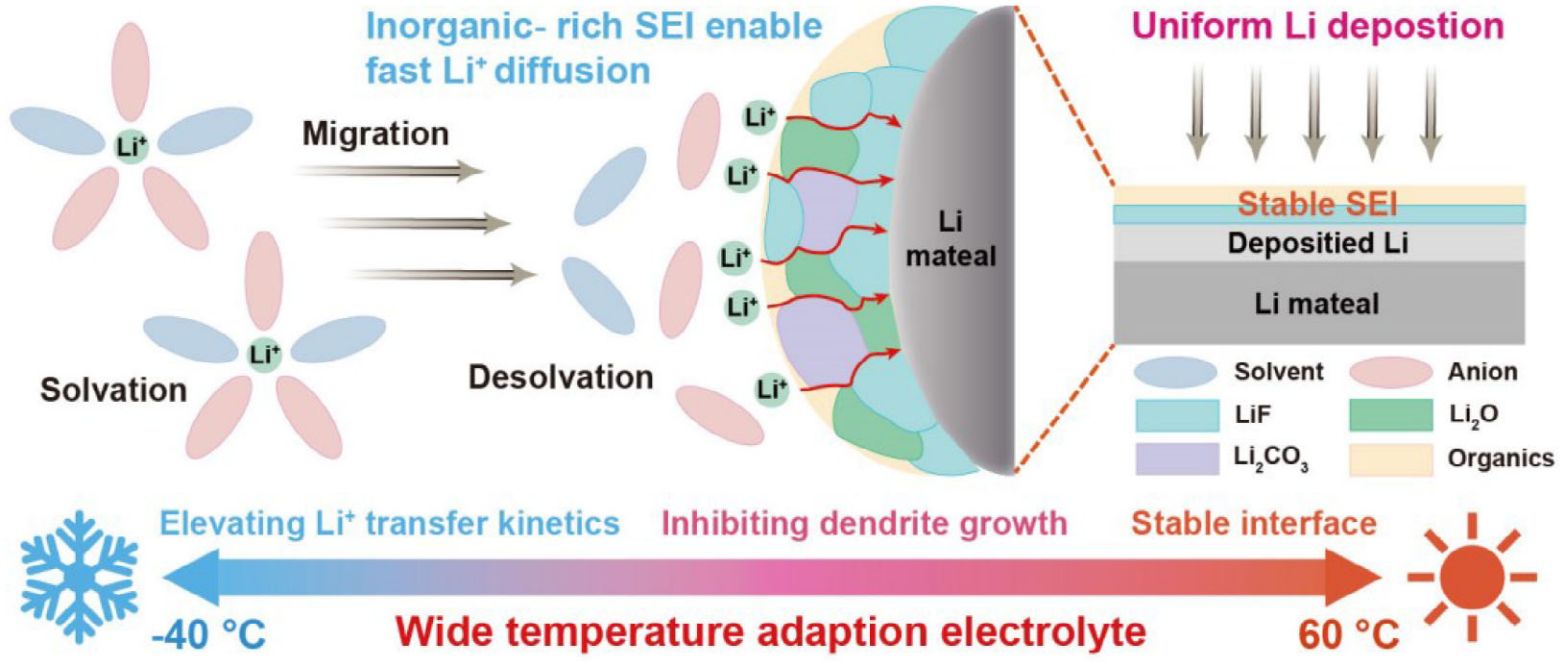
Schematics of the SEI chemistry of anion‐derived SEI.

Based on the comprehensive characterization techniques, the effect of SEI chemistry can be summarized as illustrated in Figure 6. The anion‐derived SEI facilitates the Li^+^ diffusion kinetics and suppresses the Li dendrite growth, leading to the uniform Li deposition and, thus, enabling LMBs to operate over a wide temperature range.

## Conclusion

In this work, we show that controlling the SEI chemistry plays a critical role in determining the low‐temperature performance of LMBs. Combining comprehensive characterization techniques, we found that the anion‐derived SEI with a LiF‐rich layer facilitates Li^+^ diffusion in SEI and accelerates Li^+^ desolvation at the SEI. Therefore, it promotes the kinetics of lithium metal anodes and, further, inhibits the dendrite growth at low temperatures. Consequently, WTAE enables 5.8 Ah pouch cell LMBs to operate in the wide temperature range from −40 °C to 60 °C achieving high energy density of 503.3 Wh kg^−1^ and excellent cycling for 260 cycles at 25 °C. Moreover, the 5.8 Ah pouch cell delivers ultrahigh discharge energy density of 339 Wh kg^−1^ at −40 °C. This work showcases the importance of understating the SEI chemistry to realize LMBs capable of wide temperature operation.

## Conflict of Interests

The authors declare no conflict of interest.

## Supporting information



Supporting Information S1

Supporting Video S2

## Data Availability

The data that support the findings of this study are available on request from the corresponding author. The data are not publicly available due to privacy or ethical restrictions.
