# Supplementary material for: Wide Temperature 500 Wh kg−1 Lithium Metal Pouch Cells
Source: Angew Chem Int Ed Engl. 2025 May 20;64(29):e202503693. doi: 10.1002/anie.202503693 (PMC12258678; doi:10.1002/anie.202503693)
Supplement: Supplementary file 1 — Supporting Information S1 [file ANIE-64-e202503693-s002.docx]

Wide temperature 500 Wh kg^-1^ lithium metal pouch cells

Zichun Xiao^+a^, Xu Liu^+b^, Feng Hai^a^, Yong Li^c^*, Duzhao Han^d^, Xiangwen Gao^e^*, Zhenxin Huang^a^, Yu Liu^a^, Zhen Li^a^, Wei Tang^a^*, Yuping Wu^b^*, Stefano Passerini^f,g,h^*

^a^ School of Chemical Engineering and Technology, Xi'an Jiaotong University, Xi'an, 710049, China

^b^ School of Energy and Environment & Z Energy Storage Center, Southeast University, Nanjing, 211189, China

^c^ State Key Lab Space Power Sources, Shanghai Institute Space Power Sources, Shanghai, 200245, China

^d^ CNPC Tubular Goods Research Institute (TGRI), Xi'an 710077, China

^e^ Future Battery Research Center, Global Institute of Future Technology, Shanghai Jiao Tong University, Shanghai 200240, China

^f^ Helmholtz Institute Ulm (HIU) Electrochemical Energy Storage, Helmholtzstrasse 11, 89081 Ulm, Germany

^g^ Karlsruhe Institute of Technology (KIT), P.O. Box 3640, 76021 Karlsruhe, Germany

^h^ Austrian Institute of Technology (AIT), Center for Transport Technologies, Giefinggasse 4, 1020 Wien, Austria

^+^These authors contributed equally to the work.

*Corresponding authors

***Experimental Sections***

***Materials***: Dimethyl ether (DME) and 1,1,2,2-tetrafluoroethyl-2,2,3,3- tetrafluoropropyl ether (TTE) were purchased from Duoduo Chem Co., Ltd. Lithium bis(fluorosulfonyl)amide (LiFSI) was purchased from Kelude Co., Ltd. Lithium nitrate (LiNO_3_) was purchased from Macklin. The electrolytes were prepared by dissolving 1M LiFSI into neat DME or the mixture of DME and TTE with different volume ratios.

***Characterizations***: The morphology of the deposited Li was characterized using a scanning electron microscope (MAIA3 LMH). All samples were paste on SEM stages in an Ar-filled glove box and transferred into SEM to avoid exposure to the air. The Cryo-TEM characterization was performed on Filed Emission Transmission Electron Microscope (FE-TEM, Talos-F) operated at 200 kV. The samples for Cryo-TEM characterizations were prepared by directly depositing Li metal in a TEM grid at a current density of 1 mA cm^−2^ for 0.5 h. The grid was rinsed by DME slightly twice and dried in the glove box. X-ray photoelectron spectroscopy (XPS) characterizations were performed on a PHI VersaProbe 4 by using Al anode source at 20 kV. The samples for XPS characterization were prepared by cycling for 10 cycles with a current density of 0.5 mA cm^−2^ and a capacity of 0.5 mAh cm^−2^ at -20 ℃. Raman spectroscopy was conducted on a Renishaw and a 785 nm laser source was applied. The interface evolution of Cu foil and bare Li electrode during Li plating was observed in Nikon microscope (Nikon SMZ745T) by using the in situ microscopic imaging cell (BJS-LIB-VTMS) from the Beijing SciStar technology Co. Ltd.

***Electrochemical measurements***: Coin cells (2032) were assembled in an Ar-filled glove box (O_2_<0.1 ppm, H_2_O<0.1 ppm). The separator used in coin cells was Celgard 2500. The amount of electrolyte used in each cell was 50 μL. All cycling tests were conducted on a Neware battery testing system, and all the cells were tested in a battery thermostat chamber to ensure a constant chamber regardless low temperatures or high temperatures. EIS measurements were carried out in a Princeton electrochemical work station with a frequency range of 10^-1^ to 10^6^ Hz. The EIS spectra of 1M LiFSI DME/TTE electrolytes at various temperature was obtained from Li||Li symmetric cells, and the symmetric cells were cycled for 10 cycles with a current density of 1 mA cm^−2^ and a capacity of 1 mAh cm^−2^. For CE tests, the stripping cut-off voltage is set at 0.5 V. The NCM83 electrode adopted in full cells and pouch cells was made by mixing commercial NCM83 powder, super P and PVDF in the weight ratio of 90:5:5 on Al/C foil.

For high energy density Li-pouch cells, the assembly and test of pouch cells were conducted in State Key Laboratory of Space Power-Sources, Shanghai Institute of Space Power-Sources. The cathode was made by mixing NCM811 (S85E), polyvinylidene difluoride (PVDF), Super P and VGCF with the weight ratio of 95.5:2,2:1.8:0.5 in N-methyl-2-pyrrolidone (NMP). Stacked pouch cells were assembled in a dry room with the dew point below -54 ℃ at the temperature of 20 ℃. Each pouch cell contained 13 layers of double-side cathode (the thickness of cathode current collector is 10 μm) and 14 layers double-side anode, separated by commercial separator (20 μm. Celgard 2320, Tianjing KPRT Co., Ltd), packed in Al-plastic film (73 μm, Dai Nippon Printing Co., Ltd). The pouch cells were subjected to two cycles at 0.1 C for the battery formation process. For the long-term cycling tests, the pouch cells were tested at 0.1 C for charging and 0.4 C for discharging during the cycling at 25±2 ℃. For the tests of pouch cells’ discharge energy density at -40 to 60 ℃, the pouch cells were all charged at 0.1 C and 25 ℃, then the pouch cells were discharged at 0, -20 -30, -40 and 60 ℃ at 0.1 C.

**Computational methods**: Molecular dynamic simulations were performed using the Forcite module of Materials Studio 2020. Firstly, the Amorphous Cell module was applied to construct electrolyte models by adding LiFSI, DME and TTE molecules into a cubic box based on the specific molecular ratio of our electrolytes. Condensed-phase Optimized Molecular Potentials for Atomistic Simulation Studies Ⅲ(COMPASS Ⅲ) forcefield was used to calculate molecular forces. Charge scaling factor set to 0.7. Nosé–Hoover method was used to control temperature and the Q ratio was set to 0.01. Primarily, NPT was run to minimize the energy of the system at 287 K under the pressure of 0.1 GPa. Nosé–Hoover method was used to control temperature. The cut-off radius of Van der Waals force was set to 12.5 Å. Berendsen method was used to adjust pressure. Then, the NVT process was delivered at 300 K. The simulation times of NPT and NVT are 0.5 ns and 1 ns, respectively. The time step was 1 fs. The whole NVT process was used to analyze the solvation structure.

The finite element method simulations were performed with COMSOL Multiphysics software in this study. The tertiary current distribution, Nernst–Plank interface and deformed geometry were utilized to model the Li^+^ electrochemical deposition process on the Li electrode surface with SEI layer. The boundary conditions for the electrolyte adjacent to the electrodes obey the Butler–Volmer equation for electrochemical reaction kinetics. The mass transfer in electric field follows the Nernst–Einstein Relation to obtain the mobility relating to the diffusion coefficient in electrolyte and the free convection between cathode and anode was neglected. As a model system, width and height of 200 × 200 μm^2^ was chosen. We used an initial Li ion concentration of 20 M, and the overpotential for electrochemical was set to 0.4 V with the relative equilibrium potential of 0 V. The Li ion diffusion coefficient in the electrolyte was set to 1×10−10 m^2^ s^−1^ and the Li ion diffusivity of SEI layers in the Fig. 5d and Fig. 5e is set as 100 and 50 times as sluggish as that in electrolyte, respectively.

The desolvation energy values based on the DFT were performed using Dmol^3^ package. The desolvation energy values were calculated with the equation: E = (E_a_ + E_b_) - E_a-b_, where E is the energy value, and E_a-b_ is the total energy of the relaxed a and b models at the equilibrium state. E_a_ and E_b_ are the self-consistent field (SCF) calculation energy values of geometry-optimized a and b models. Electron exchange correlation was constructed by Perdew-Burke-Ernzerhof (PBE) function with generalized gradient approximation (GGA). All calculations were converged with energy change <10^-5^ Ha and force <0.004 Ha/Å.

***Supplementary Figures***


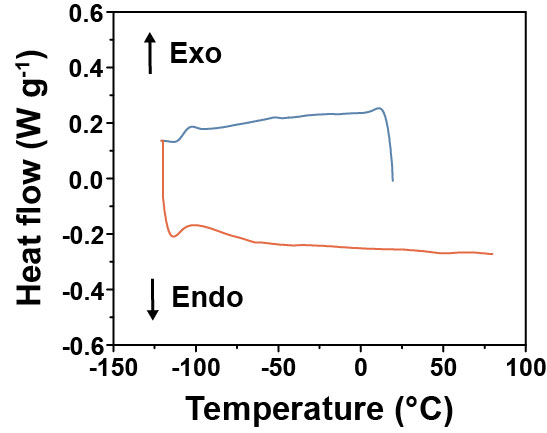


**Figure S1.** Differential scanning calorimetry (DSC) experiment of WTAE in the temperature range of −120 to 80 ℃.


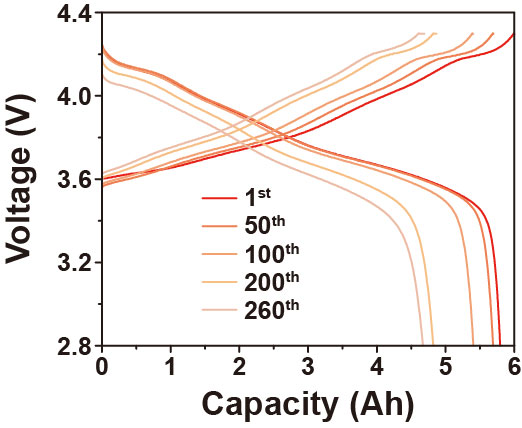


**Figure S2.** Selected voltage-capacity curves of a 5.8 Ah pouch cell.


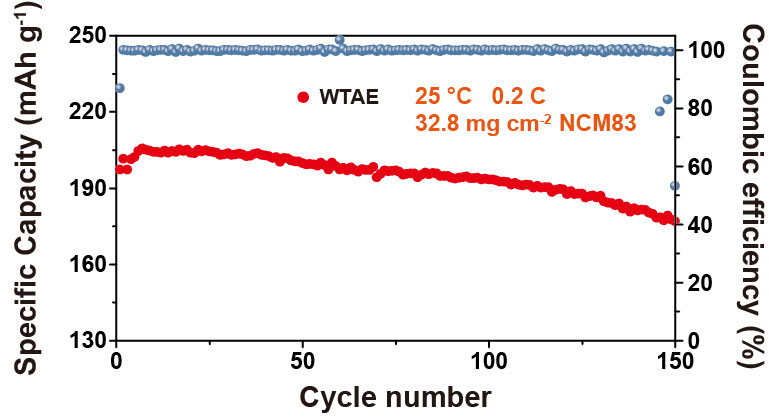


**Figure S3.** Long-term cycling performance of ultra-high cathode mass loading of Li||NCM83 coin cells using WTAE at 0.2 C and 25 ℃.

Li||NCM83 coin cells with WTAE stably cycled for 150 cycles at 0.2 C and 25 ℃, maintaining a capacity retention of 89.7%.


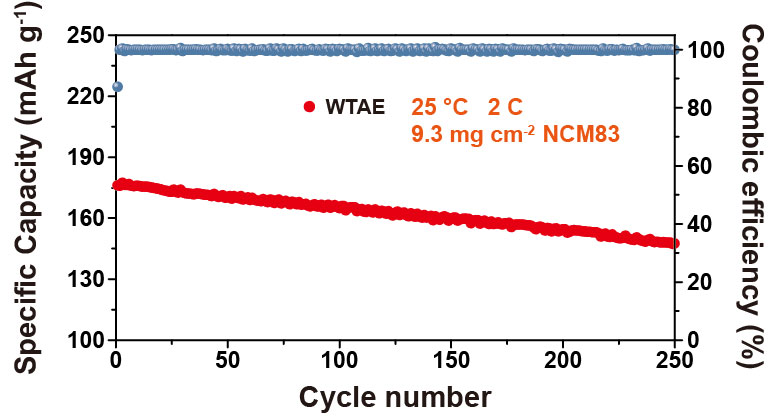


**Figure S4.** Long-term cycling performance of Li||NCM83 coin cells using WTAE at 2 C and 25 ℃.

Li||NCM83 coin cells with WTAE stably cycled for 250 cycles at 2 C and 25 ℃, maintaining a capacity retention of 83.8%.


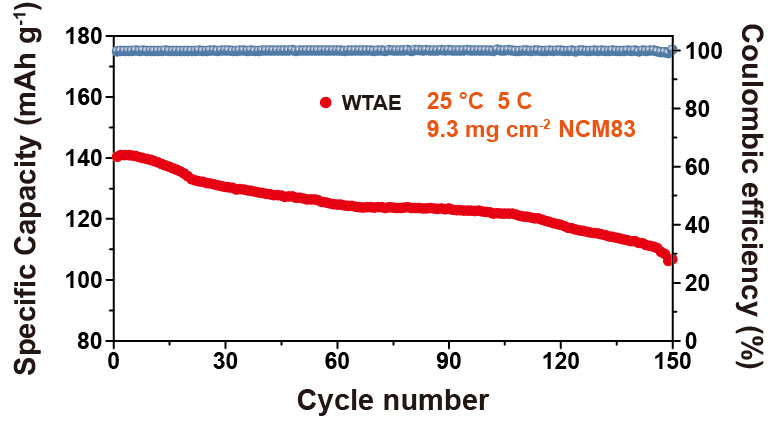


**Figure S5.** Long-term cycling performance of Li||NCM83 coin cells using WTAE at 5 C and 25 ℃.

Even at a high rate of 5 C, Li||NCM83 coin cells with WTAE stably cycled for 150 cycles, maintaining a capacity retention of 76.2%.


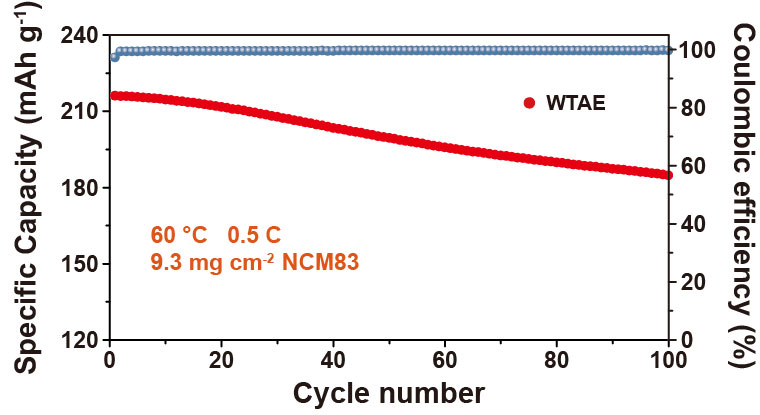


**Figure S6.** Long-term cycling performance of Li||NCM83 coin cells using WTAE at 0.5 C and 60 ℃.

At high temperature, i.e., 60 ℃, Li||NCM83 coin cells with WTAE stably cycled for 100 cycles at 0.5 C, maintaining a capacity retention of 85.5%.


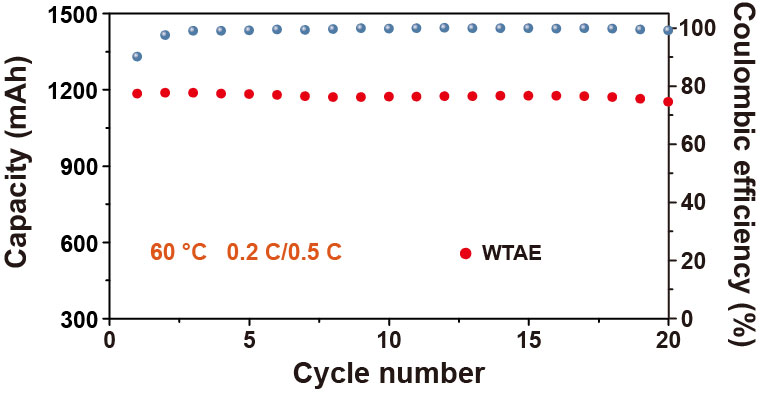


**Figure S7.** Cycling performance of 1.1 Ah pouch cells using WTAE at 0.2 C/0.5 C and 60 ℃.

At 60 ℃, the 1.1 Ah pouch cells with WTAE stably cycled for 20 cycles at 0.2 C/0.5 C, maintaining a capacity retention of 97.2%. The stable cycling of coin cells and pouch cells proves that WTAE enables LMBs to operate at a high temperature (60 ℃).


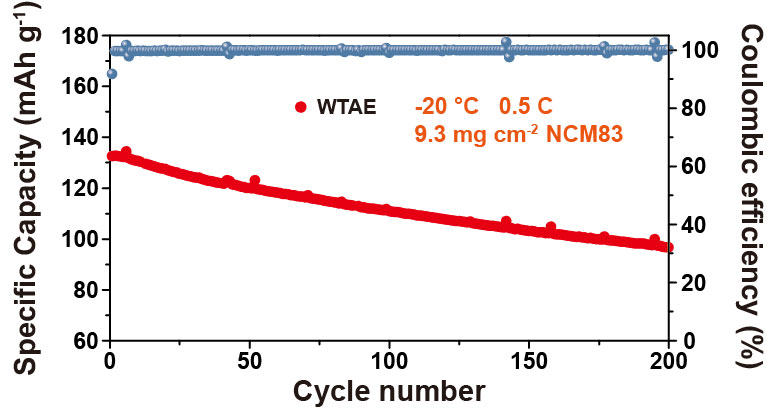


**Figure S8.** Long-term cycling performance of Li||NCM83 coin cells using WTAE at 0.5 C and -20 ℃.

At a low temperature (-20 ℃), Li||NCM83 coin cells with WTAE stably cycled for 200 cycles at 0.5 C, maintaining a capacity retention of 72.9%. The stable cycling proves that WTAE enables LMBs to operate at -20 ℃


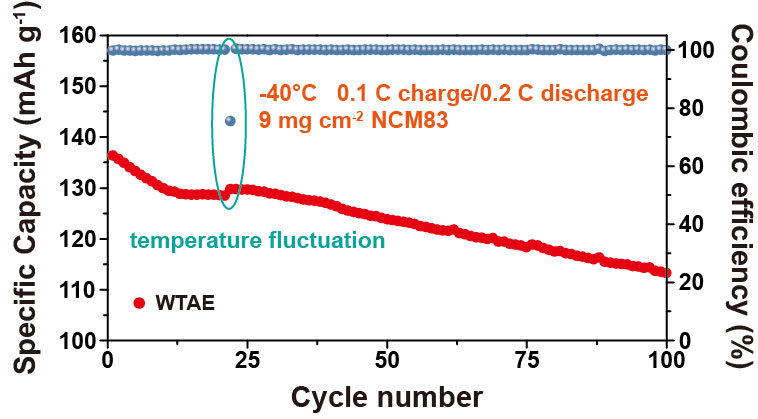


**Figure S9.** Long-term cycling performance of Li||NCM83 coin cells using WTAE at 0.1 C charge/0.2 C discharge and -40 ℃.

At very low temperature, -40 ℃, Li||NCM83 coin cells with WTAE stably cycled for 100 cycles at 0.1 C charge/0.2 C discharge, maintaining a capacity retention of 83.1%. The stable cycling proves that WTAE enable LMBs to operate at such a low temperature (-40 ℃).

Combining all results of pouch cells and coin cells electrochemical performance, demonstrates that WTAE enables LMBs with stable cycling in the wide temperature range from -40℃ to 60℃.


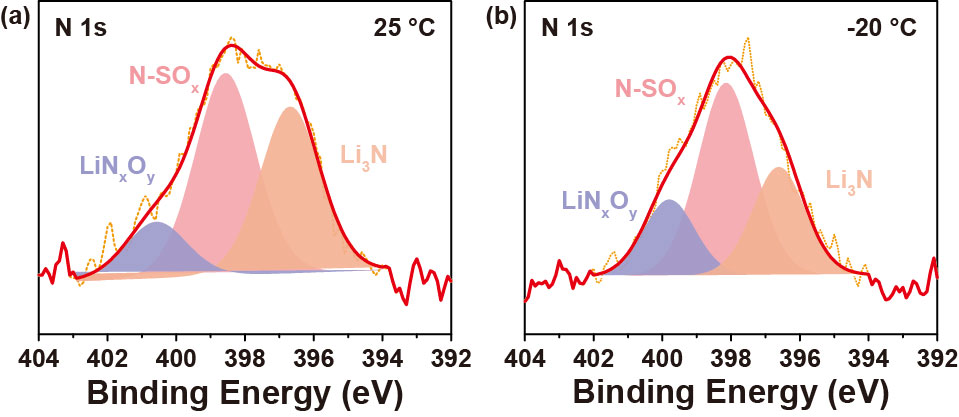


**Figure S10.** N 1s spectra of Li foil cycled in WTAE at a) 25 ℃; b) -20 ℃.

It can be observed that Li_3_N and LiN_x_O_y_ exist in the SEI formed in the WTAE at 25 and -20 ℃. The high ionic conductivity of Li_3_N and LiN_x_O_y_ in the SEI facilitates fast Li^+^ diffusion, leading to uniform and rapid Li deposition.


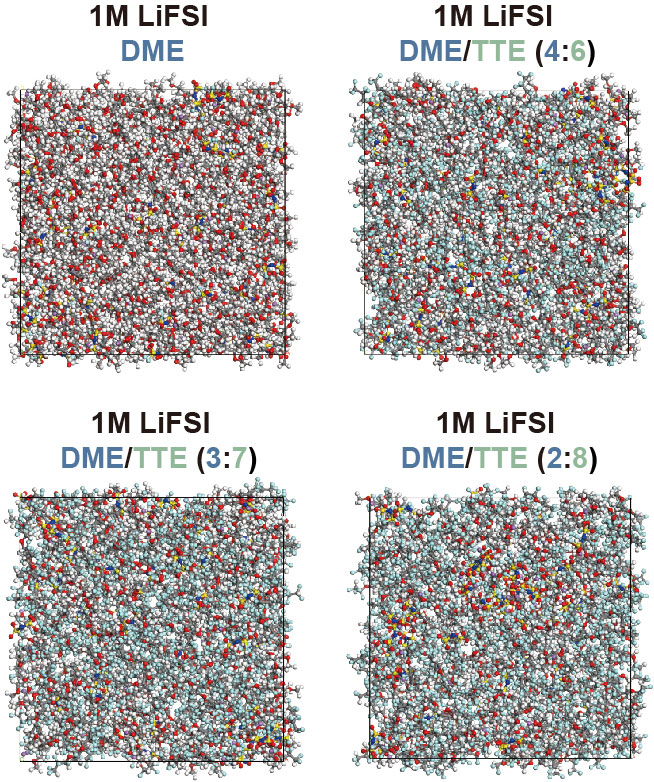


**Figure S11.** Snapshots of MD simulation in different electrolytes.


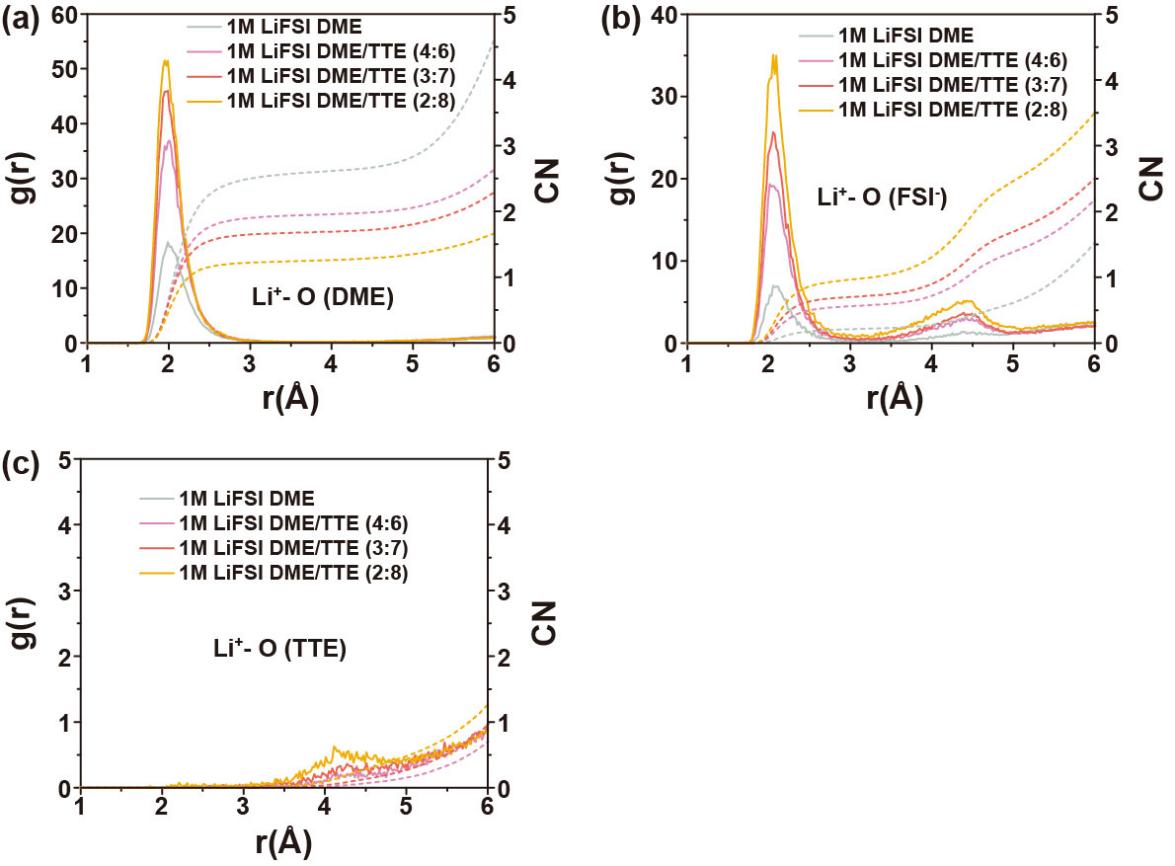


**Figure S12.** Radial distribution functions and coordination numbers of a) DME, b) FSI^-^ oxygens and c) TTE with respect to Li^+^.

It can be observed that increasing the TTE content, the coordination number of DME gradually decreases while that of FSI^-^ increases, proving that the Li^+^ coordination gradually transforms from solvent-dominated to anion-dominated.


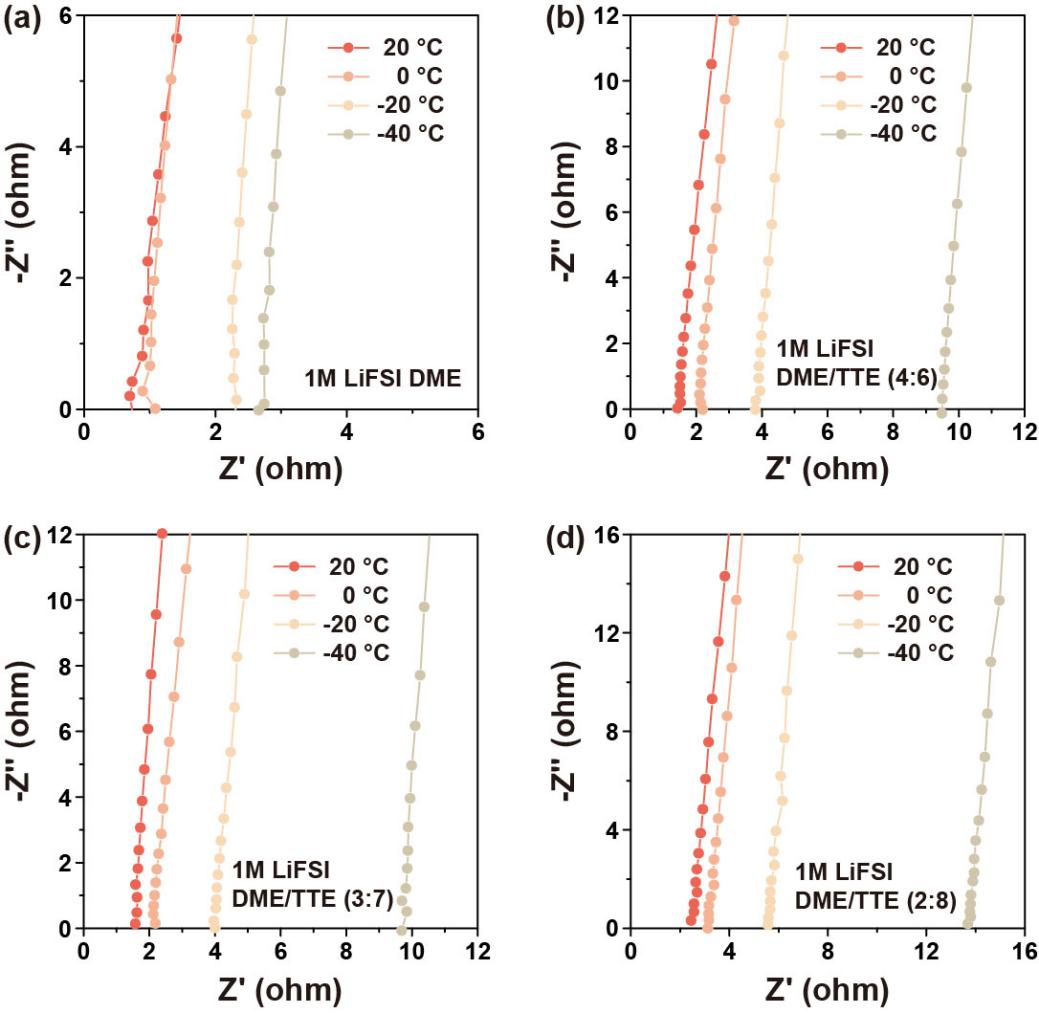


**Figure S13**. EIS spectra of symmetric coin cells with stainless steel electrodes at various temperatures. (a) 1M LiFSI DME; (b) 1M LiFSI DME/TTE (4:6); (c) 1M LiFSI DME/TTE (3:7); (d) 1M LiFSI DME/TTE (2:8).


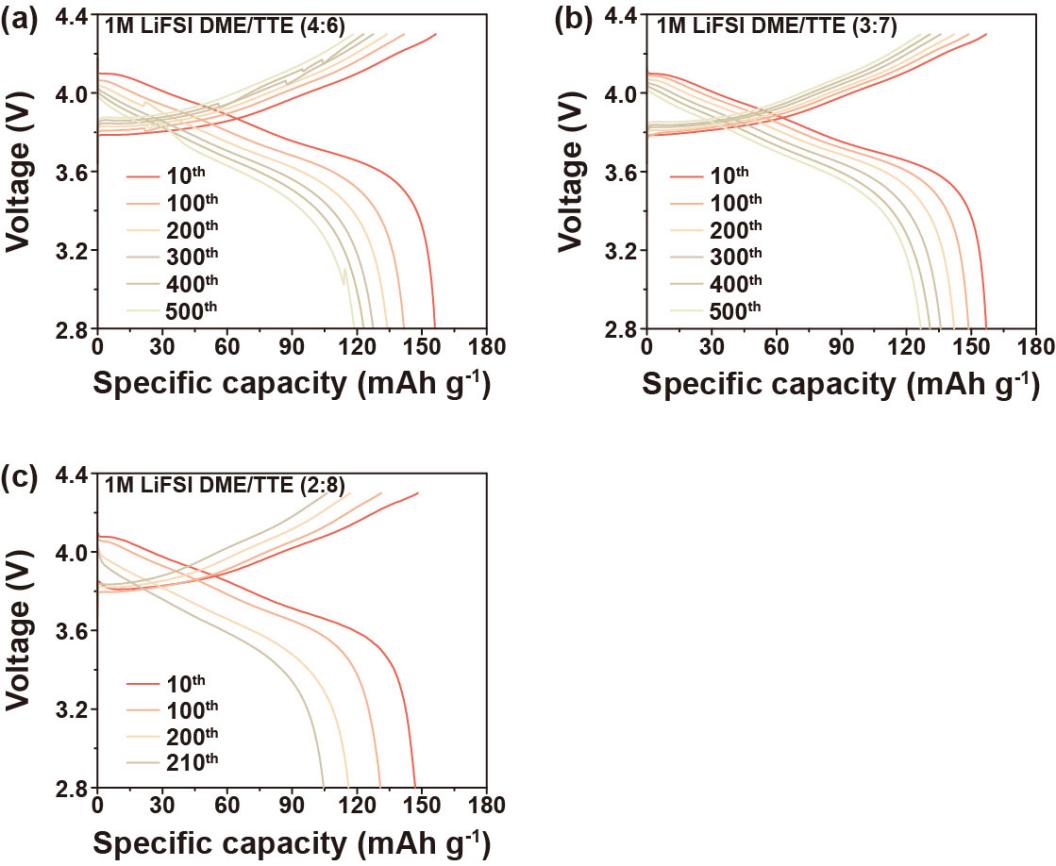


**Figure S14.** a)-c) Discharge/charge curves of Li||NCM83 cells using 1M LiFSI DME/TTE electrolytes at -20℃.


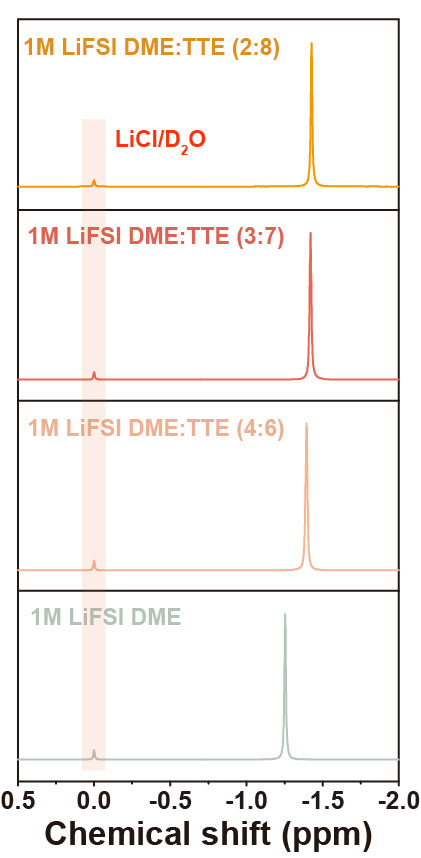


**Figure S15.** ^7^Li NMR spectra of 1M LiFSI DME/TTE electrolytes.


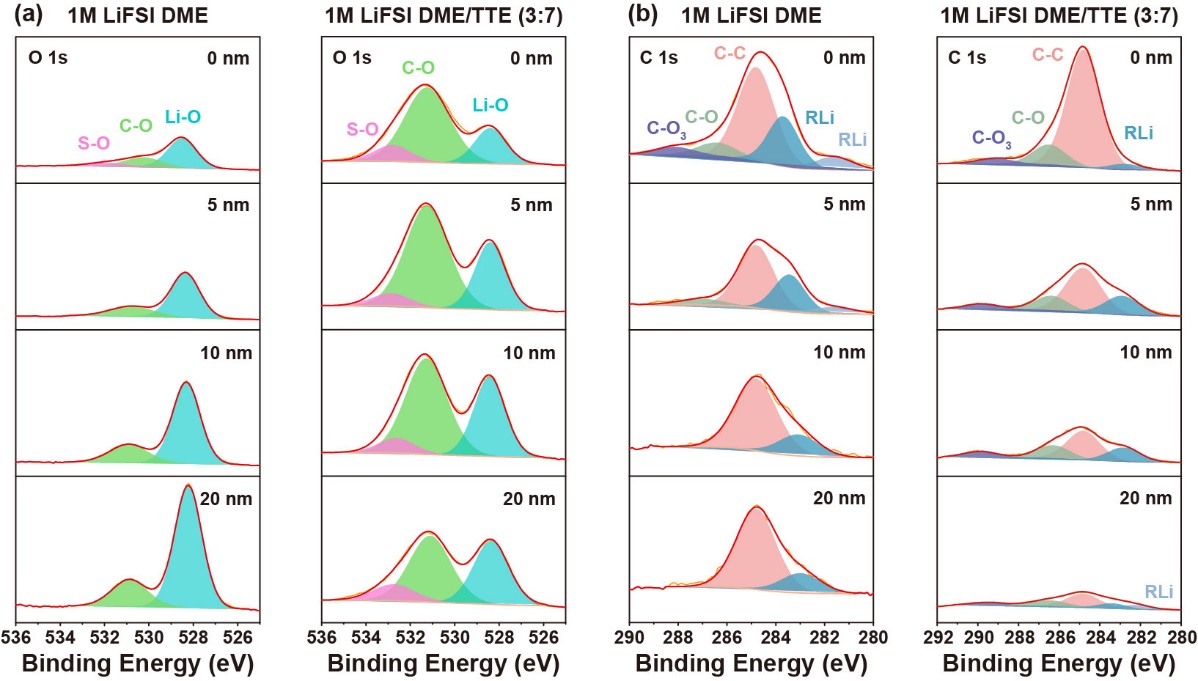


**Figure S16.** XPS spectra for a) O 1S and b) C 1S with different Ar^+^ sputtering depth on Li foils in 1M LiFSI DME and 1M LiFSI DME/TTE (3:7) electrolytes.


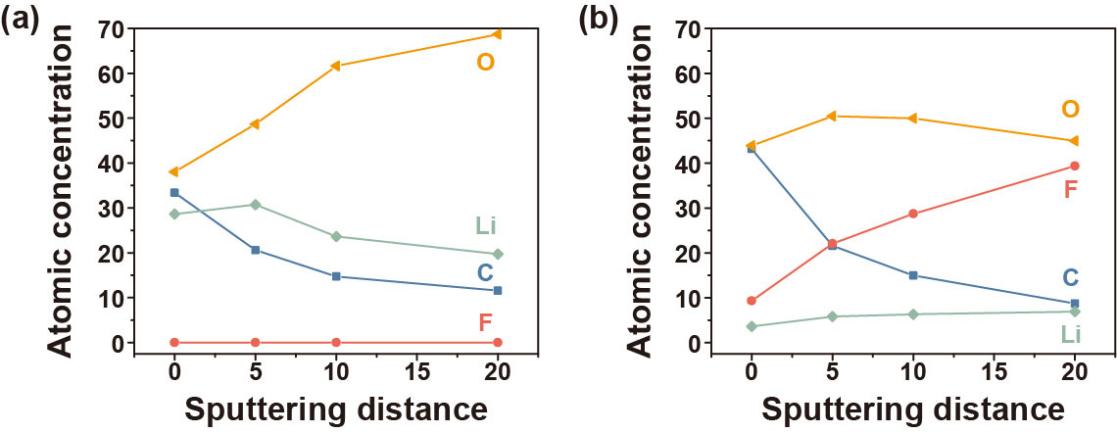


**Figure S17.** Quantified atomic ratios of the elements in SEI formed in a) 1M LiFSI DME and b) 1M LiFSI DME/TTE (3:7)


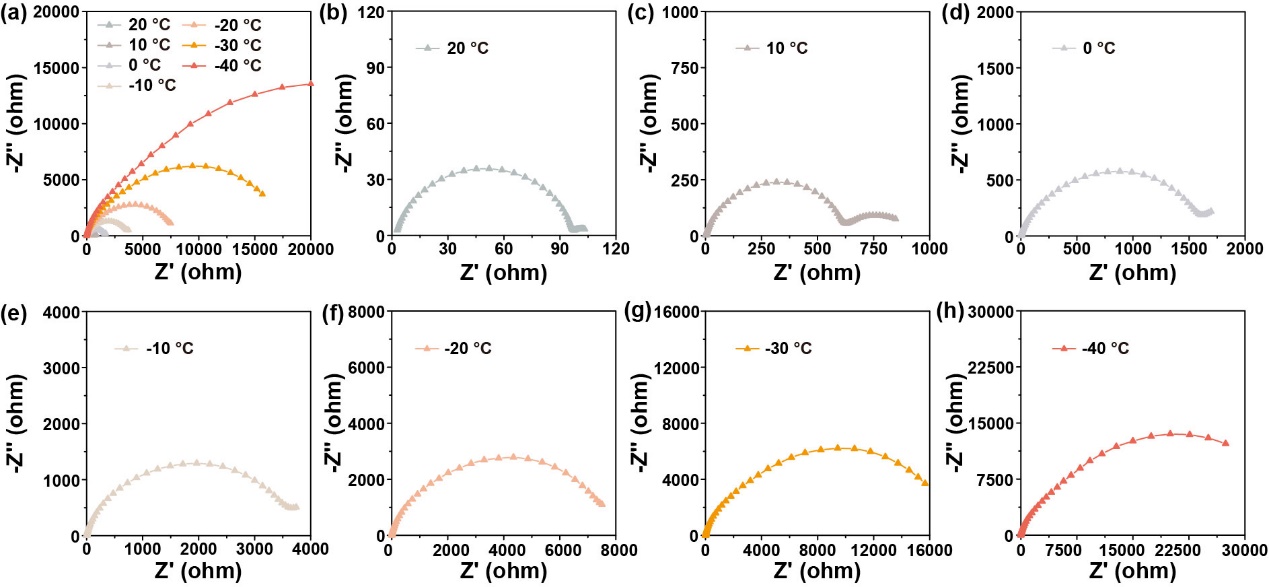


**Figure S18.** EIS spectra of Li||Li symmetric cells using 1M LiFSI DME electrolyte at temperature ranging from -40 ℃ to 20 ℃.


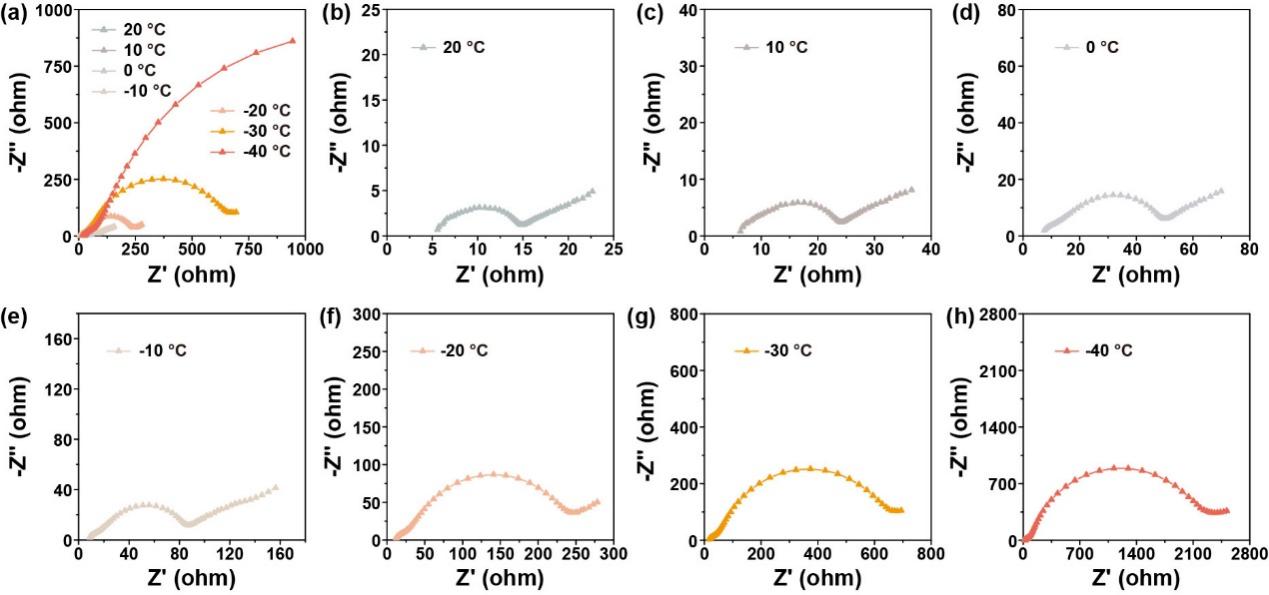


**Figure S19.** EIS spectra of Li||Li symmetric cells using 1M LiFSI DME/TTE (4:6) electrolyte at temperature ranging from -40 ℃ to 20 ℃.


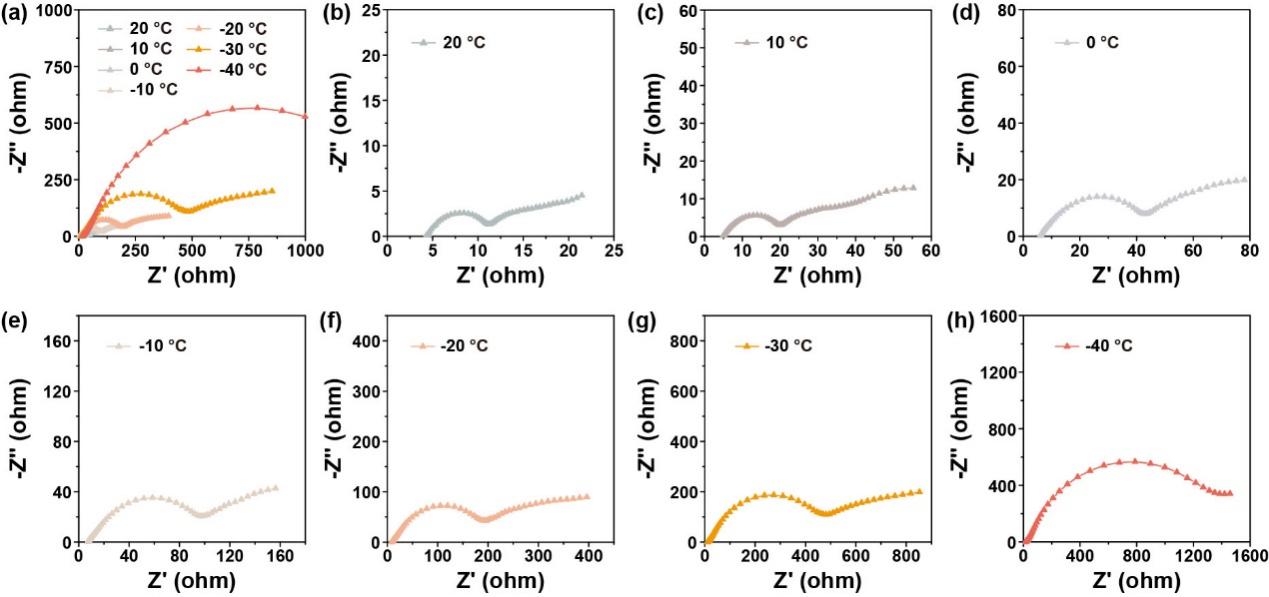


**Figure S20.** EIS spectra of Li||Li symmetric cells using 1M LiFSI DME/TTE (3:7) electrolyte at temperature ranging from -40 ℃ to 20 ℃.


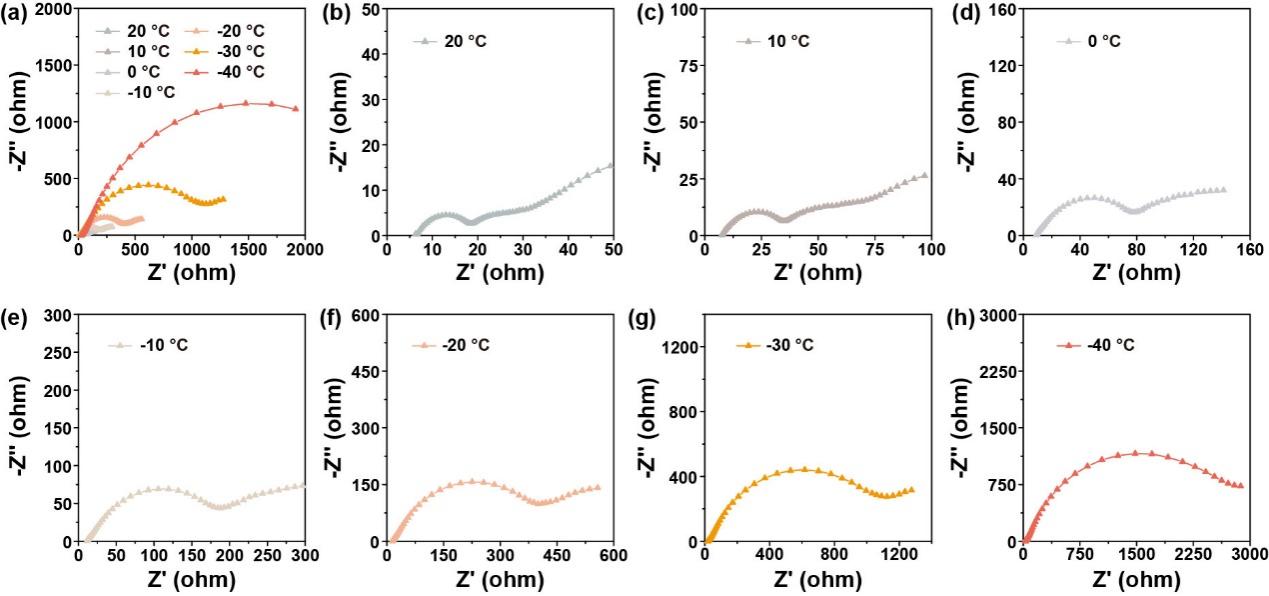


**Figure S21.** EIS spectra of Li||Li symmetric cells using 1M LiFSI DME/TTE (2:8) electrolyte at temperature ranging from -40 ℃ to 20 ℃.


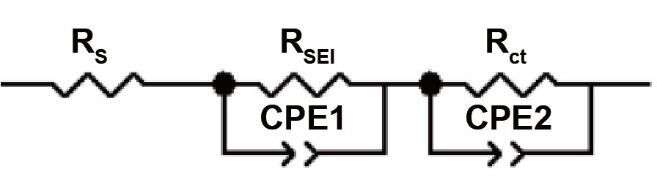


**Figure S22.** Equivalent circuit used for the fit of the EIS data.


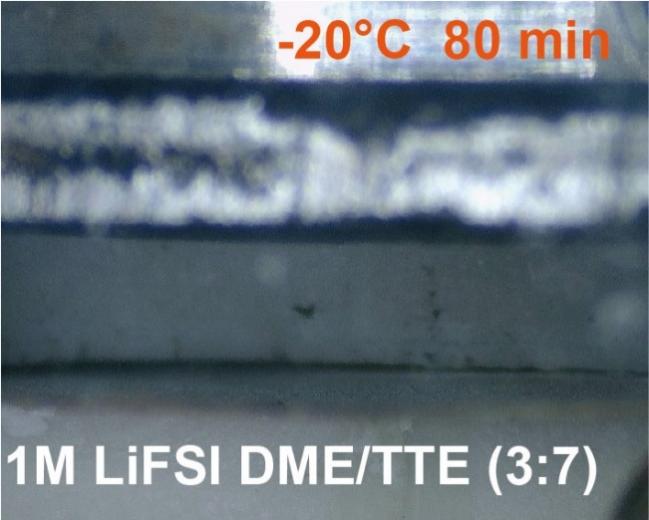


**Figure S23.** In-situ optical image of the Li-Cu cell with 1M LiFSI DME/TTE (3:7) after Li plating on Cu for 80 min.


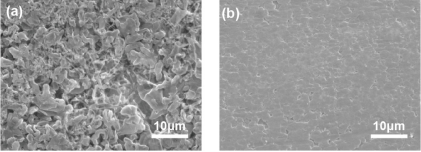


**Figure S24.** SEM images depicting the morphology of 5mAh cm^-2^ Li deposit at -20 ℃ in Li-Cu cells employing a)1M LiFSI DME and b) 1M LiFSI DME/TTE (3:7).
